# Supplementary material for: Novel Yellow Azo Pyridone Derivatives with Different Halide Atoms for Image-Sensor Color Filters
Source: Molecules. 2022 Oct 5;27(19):6601. doi: 10.3390/molecules27196601 (PMC9572834; doi:10.3390/molecules27196601)
Supplement: Supplementary file 1 [file molecules-27-06601-s001.zip › molecules-1911844-supplementary.pdf]

Novel Yellow Azo Pyridone Derivatives with Different Halide Atoms for Image-Sensor Color Filters

Sunwoo Park[a,1], Yuna Kang[a,1], Hyukmin Kwon[a], Seokwoo Kang[a], Hayoon Lee[a], Chun Yoon[b] and Jongwook Park[a,*]

[a]Integrated Engineering, Department of Chemical Engineering, Kyung Hee University, Yongin 17104, Korea
[b]Department of Chemistry, Sejong University, 98 Gunja-dong, Gwangjin-gu, Seoul 143-747, Korea
**E-mail: jongpark@khu.ac.kr*

[1]Sunwoo Park and Yuna Kang contributed equally to this work as first authors.

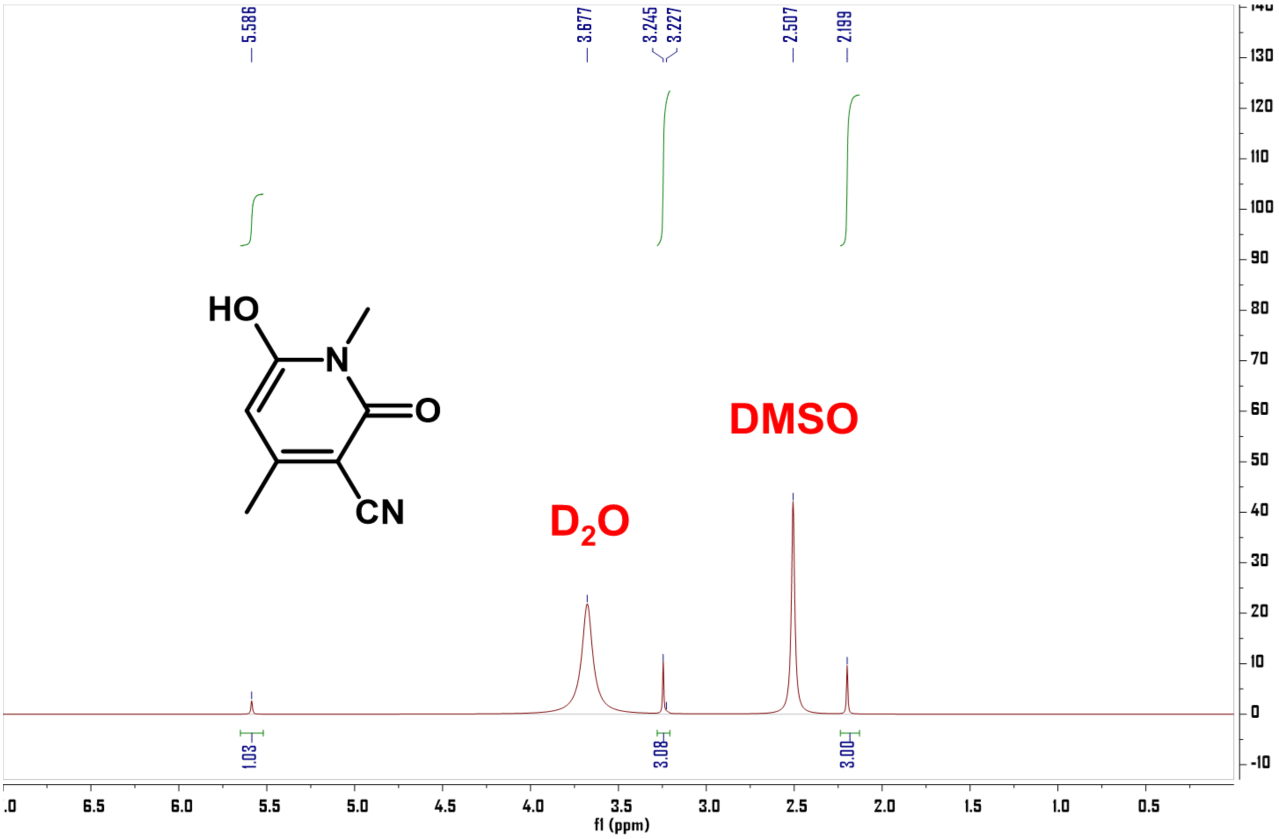

Figure S1. ^{1}H NMR spectra of 6-hydroxy-1,4-dimethyl-2-oxo-1,2-dihydropyridine-3-carbonitrile

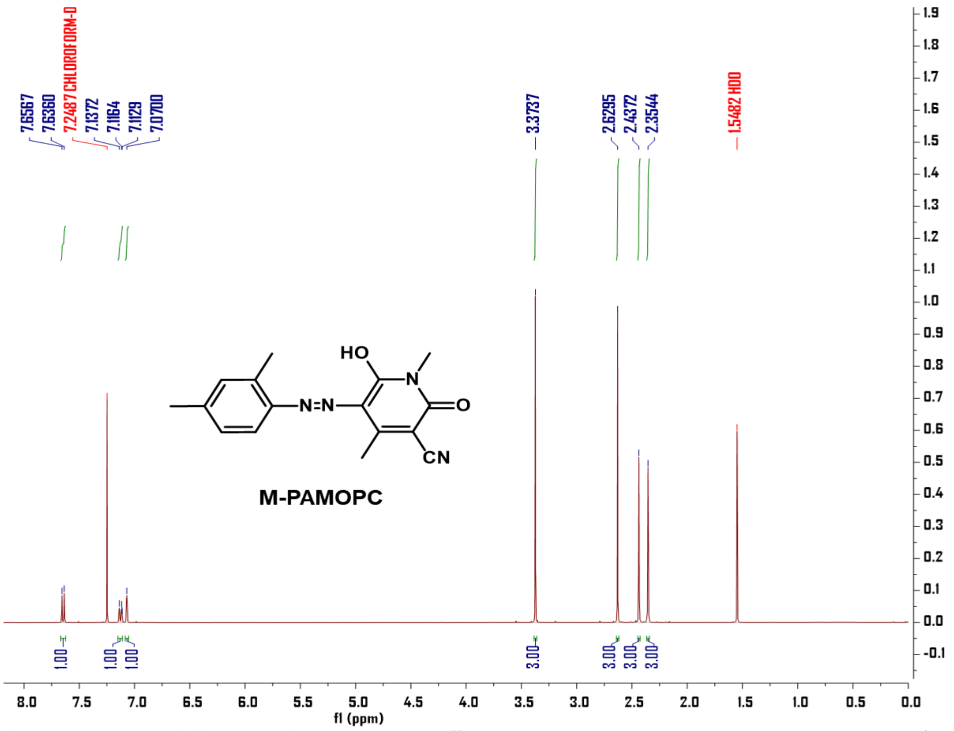

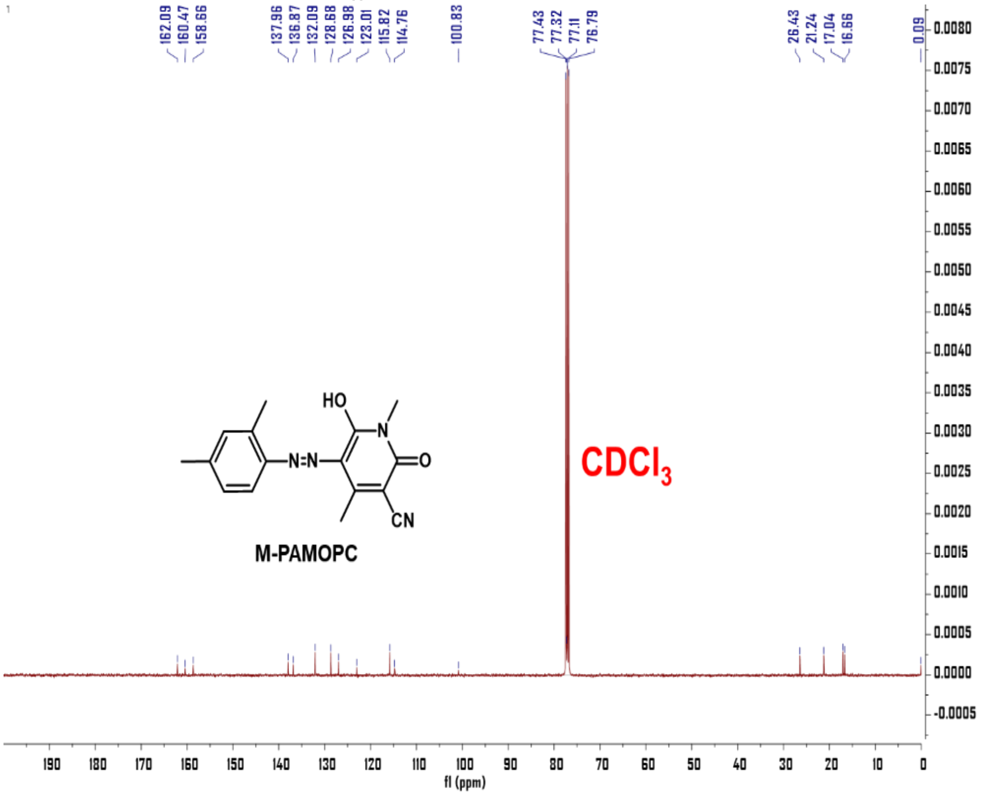

Figure S2. ^{1}H NMR(up) and ^{13}C NMR (down) spectra of M-PAMOPC

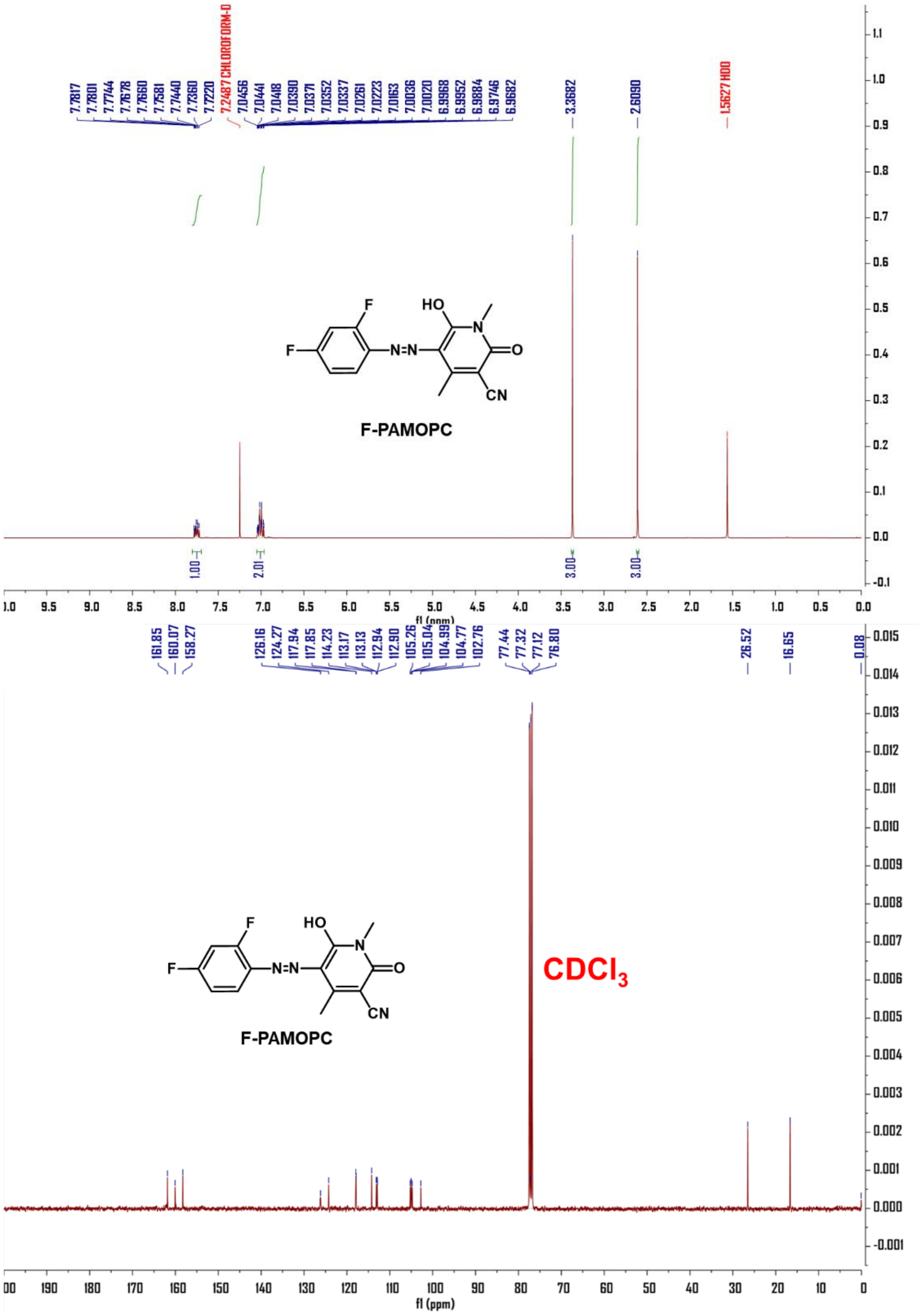

Figure S3. ^{1}H NMR(up) and ^{13}C NMR (down) spectra of F-PAMOPC

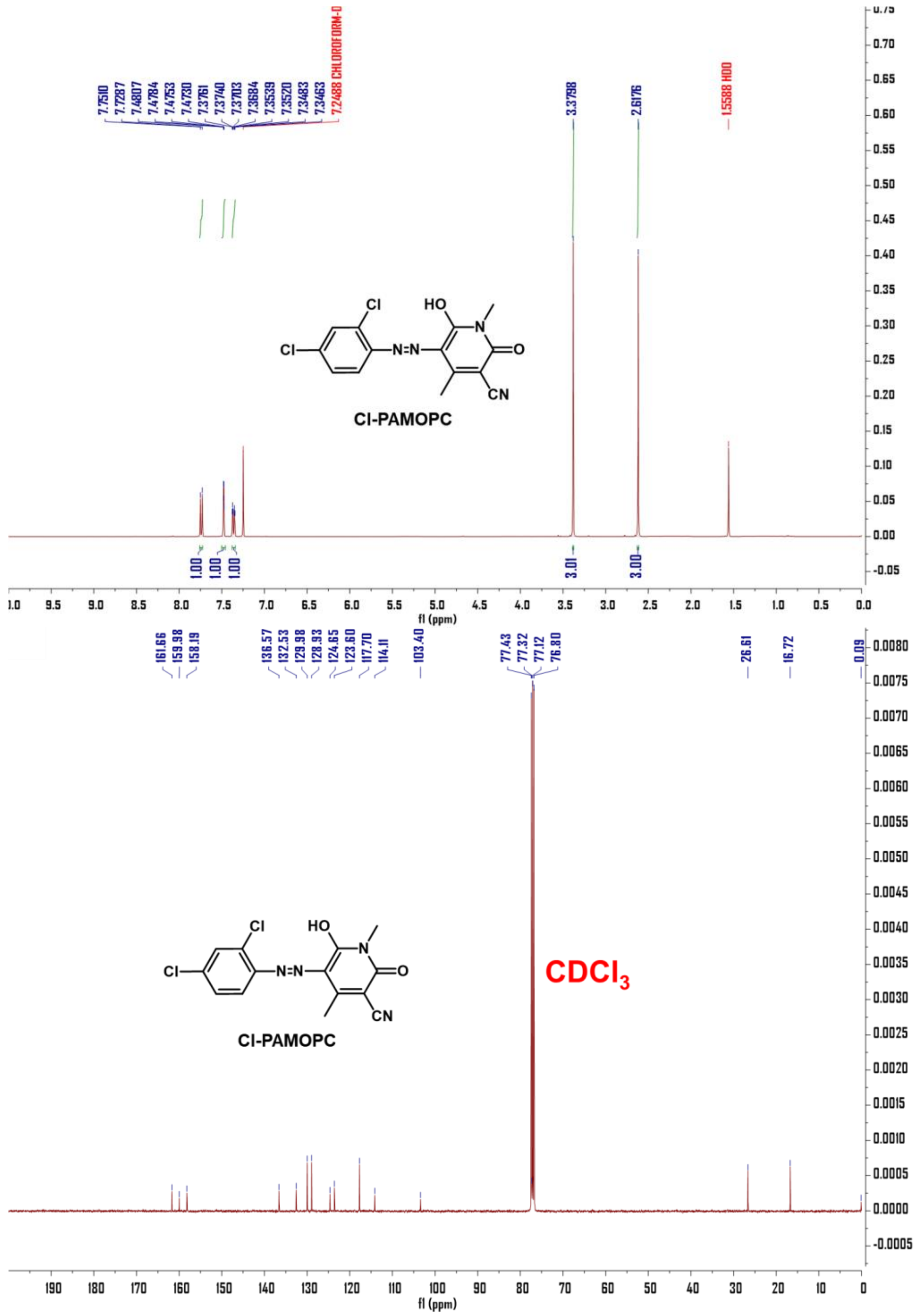

Figure S4. ^{1}H NMR(up) and ^{13}C NMR (down) spectra of Cl-PAMOPC

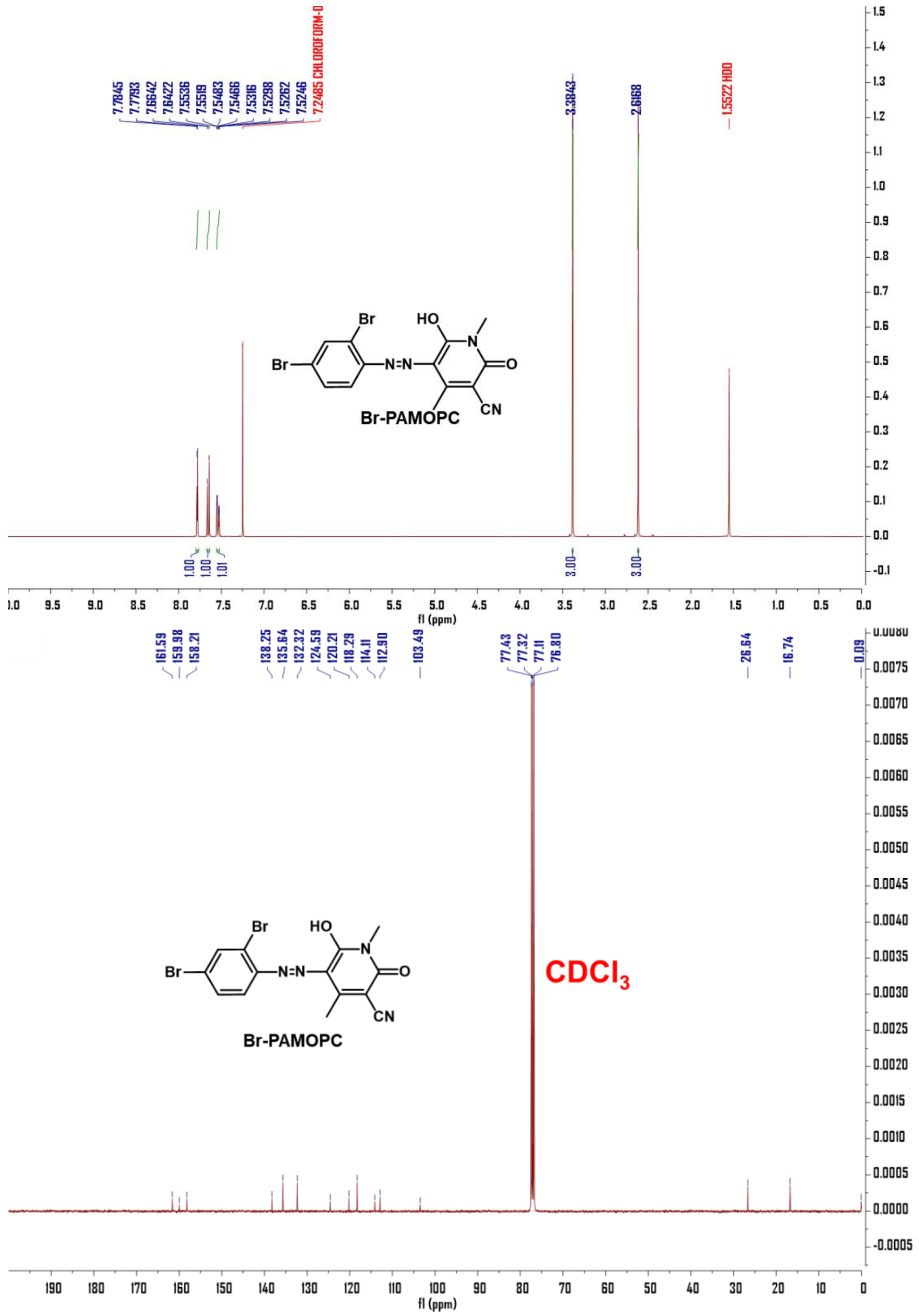

Figure S5. ^{1}H NMR(up) and ^{13}C NMR (down) spectra of Br-PAMOPC

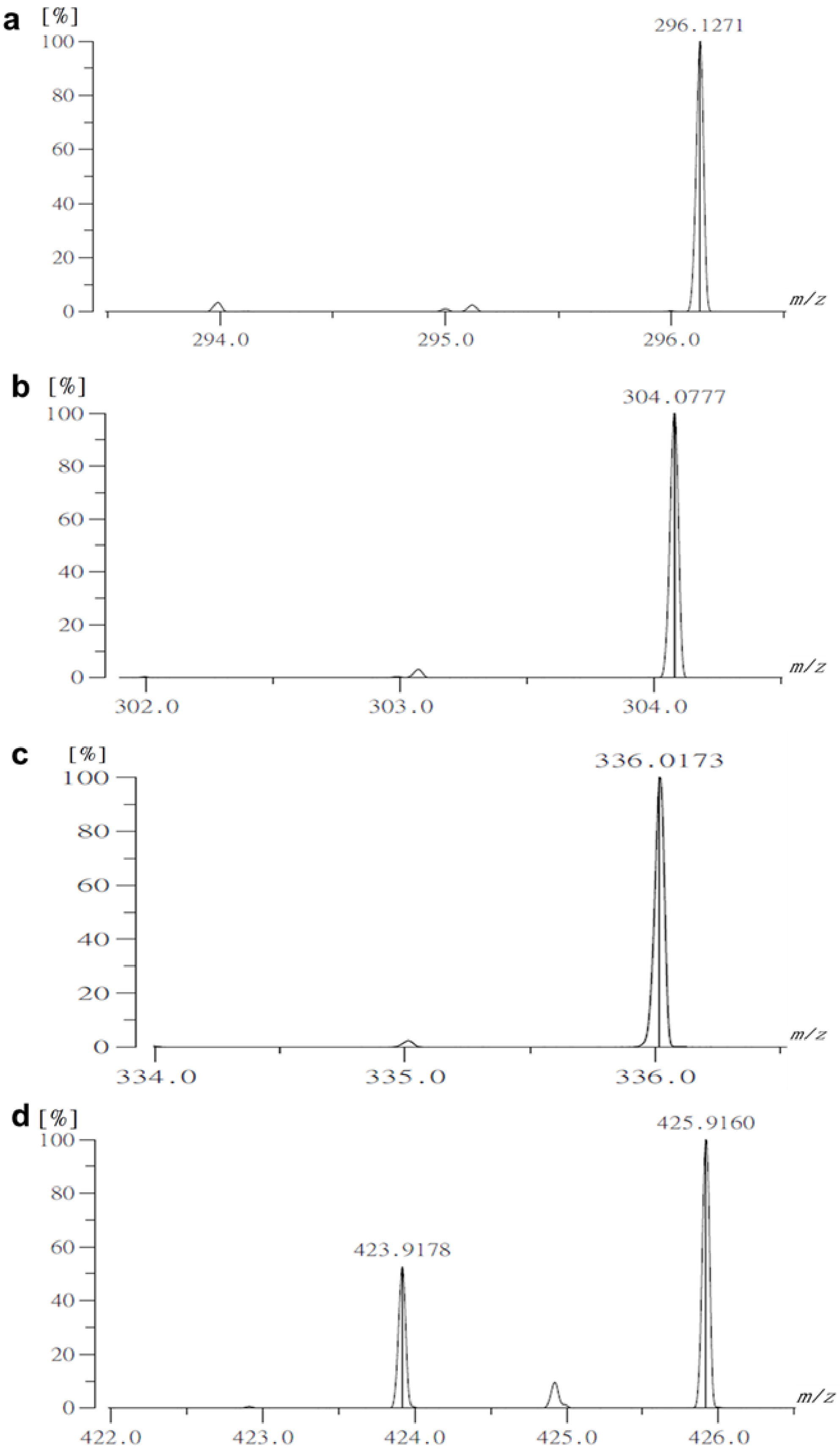

Figure. S6. high resolution mass spectroscopy (HR-MS) spectra of (a) M-PAMOPC, (b) F-PAMOPC, (c) Cl-PAMOPC, and (d) Br-PAMOPC

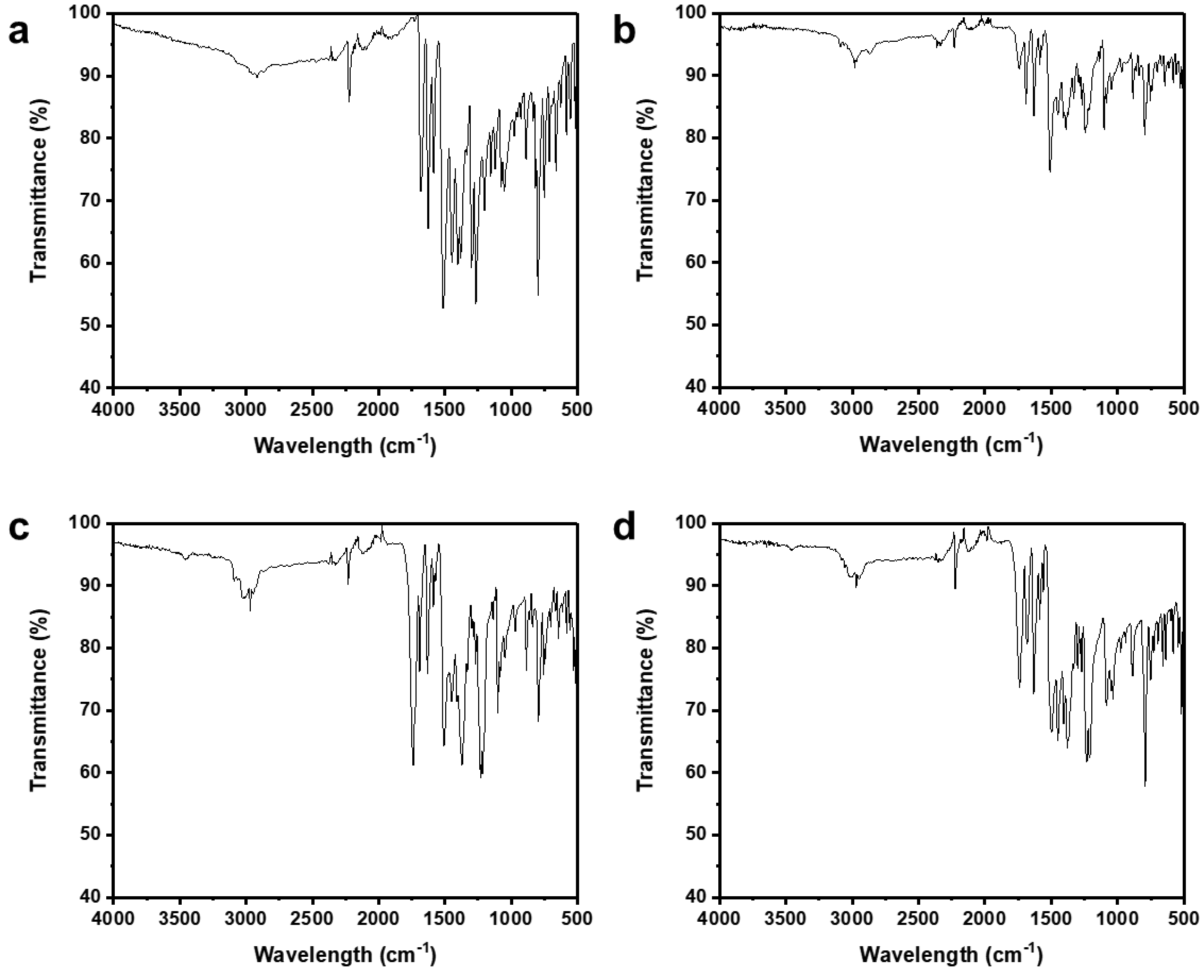

Figure S7. Fourier transform infrared spectroscopy (FTIR) spectra of (a) M-PAMOPC, (b) F-PAMOPC, (c) Cl-PAMOPC, and (d) Br-PAMOPC

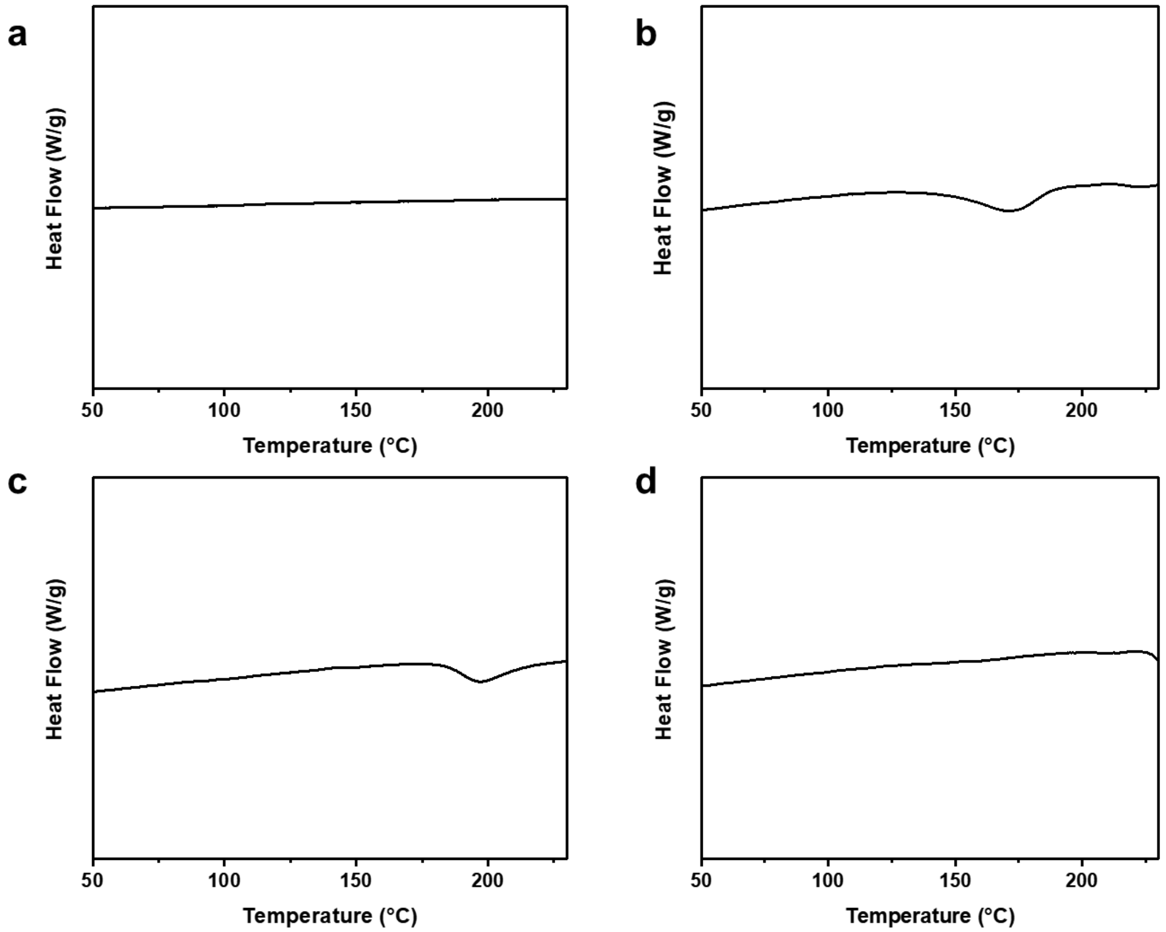

Figure S8. Differential scanning calorimetry (DSC) curves of (a) M-PAMOPC, (b) F-PAMOPC, (c) Cl-PAMOPC, and (d) Br-PAMOPC

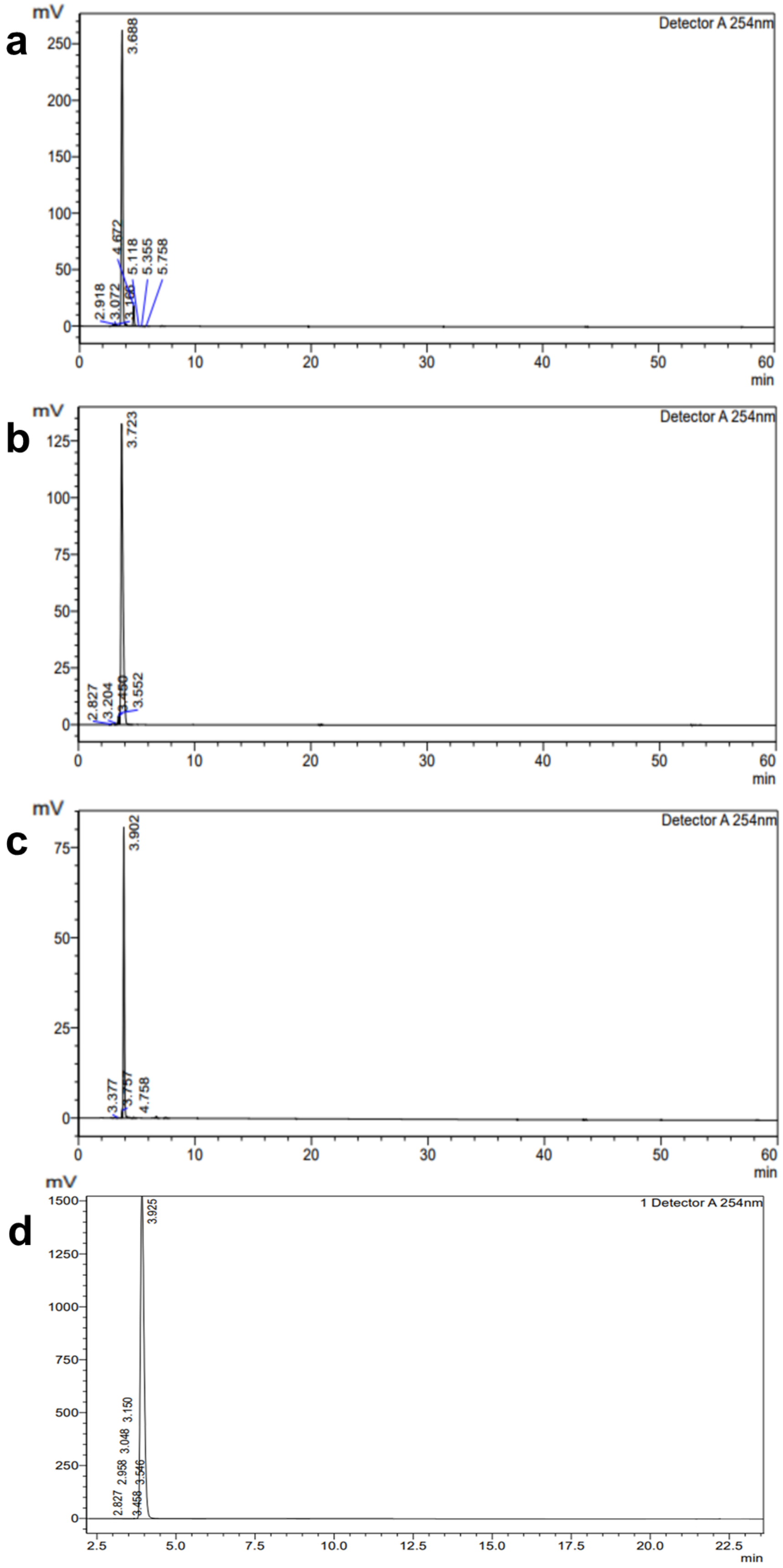

Figure S9. High performance liquid chromatography (HPLC) chromatograms of (a) M-PAMOPC, (b) F-PAMOPC, (c) Cl-PAMOPC, and (d) Br-PAMOPC
